# Anti-inflammatory effects of α-humulene on the release of pro-inflammatory cytokines in lipopolysaccharide-induced THP-1 cells

**DOI:** 10.1007/s12013-024-01235-7

**Published:** 2024-02-22

**Authors:** Lucas Becker, Dirk Holtmann

**Affiliations:** 1 Bioprocess Intensification, Institute of Bioprocess Engineering and Pharmaceutical Technology, University of Applied Sciences Mittelhessen, Wiesenstrasse 14, 35390 Giessen, Germany; 2 Institute of Process Engineering in Life Sciences, Karlsruhe Institute of Technology, Fritz-Haber-Weg 4, 76131 Karlsruhe, Germany

**Keywords:** α-humulene, Anti-inflammatory, Cytokines, THP-1 cells, Lipopolysaccharides, Inflammation

## Abstract

While acute inflammation is an essential physical response to harmful external influences, the transition to chronic inflammation is problematic and associated with the development and worsening of many deadly diseases. Until now, established pharmaceutical agents have had many side effects when used for long periods. In this study, a possible anti-inflammatory effect of the sesquiterpene α-humulene on lipopolysaccharide (LPS) induction was tested. Herein, human THP-1-derived macrophages were used and their pro-inflammatory interleukin-6 (IL-6), tumor necrosis factor alpha (TNF-α), and interleukin-1β (IL-1β) cytokine release was measured by means of enzyme-linked immunosorbent assay. A dose-dependent effect of α-humulene on IL-6 release was observed at 0.5 and 100 µM α-humulene, with a maximum IL-6 inhibition of 60% compared to the LPS reference value after the addition of 100 µM α-humulene. TNF-α as well as IL-1β cytokine concentrations were not reduced by the addition of 0.5 and 100 µM α-humulene. This study suggests that α-humulene has potential as a promising natural alternative to established pharmaceuticals for the treatment of elevated IL-6 levels and chronic inflammation in humans.

## Introduction

Inflammation can be divided into two types: acute inflammation and persistent chronic inflammation. Acute inflammation is an important physical repair mechanism and a life-sustaining response to extrinsic influences, such as injuries and viral or bacterial infections [1]. For acute inflammatory reactions, common anti-inflammatory drugs from the group of corticosteroids or non-steroidal anti-inflammatory drugs (NSAID) such as aspirin or ibuprofen can be used [2]. Chronic inflammation, however, exceeds the benefits of acute inflammation due, to its continuous stimulation of the organism, and can lead to a wide range of physical and mental diseases, from diabetes and Alzheimer’s to cancer [1, 3, 4]. Diseases that are considered to be the consequence of chronic inflammation currently account for more than 50% of the world’s deaths [5, 6]. A long-term use of NSAID for the treatment of chronic inflammation, though, can cause many undesirable side effects, suppresses the patient’s immune system, and damages the liver and kidney, thus worsening symptoms and overall physical health [7].

In the transition from acute to chronic inflammation, the sustained increased release of pro-inflammatory cytokines and the resulting sustained imbalance with anti-inflammatory cytokines play an important role [8]. Thus, there is an urgent need for new anti-inflammatory agents with fewer side effects that counteract the release of pro-inflammatory cytokine. Therefore, drugs based on plant extracts or their pharmacologically active ingredients could be a promising alternative, which allow for longer medication periods and results in fewer side effects compared to classic anti-inflammatory pharmaceuticals. Plant extracts have been used in traditional medicine for thousands of years, and studies are increasingly demonstrating their anti-inflammatory effects [9]. Due to the composition of various active secondary plant compounds, such as terpenes, essential oils can cause a remodulation and influence the nuclear factor kappa-light-chain-enhancer of activated B cells (NF-κB) signaling pathway, which is significantly involved in the inflammatory cascade through the transcription and release of pro-inflammatory cytokines, such as TNF-α, IL-1, IL-6, and IL-8 [10, 11]. The anti-inflammatory effect of plant components could also be observed after administering lipopolysaccharides (LPS) in the murine microglial cell line BV-2. Thyme essential oil or its major monoterpene components, linalool, geraniol, and thujanol, reduced LPS-induced IL-6 and TNF-α release [12]. In this context, the administration of bacterial LPS, which are bacterial toxins and trigger signaling cascades of inflammatory responses, initiates the bacterial inflammatory process [13]. This study focuses on the sesquiterpene α-humulene, which, along with its isomer β-caryophyllene, is found in many plants around the world. The compound is named after its main source, the hop plant *Humulus lupulus* [14, 15].

In addition to its aroma and odor properties, α-humulene has promising pharmacological effects, including anti-inflammatory, anti-microbial, anti-biofilm, and anti-fungal. It also has anti-cancer properties, such as chemosensitizing and growth inhibition [16–19]. To demonstrate the potential anti-inflammatory effects of α-humulene and its transfer to human cells, the human THP-1 cell line was chosen as a comparative model. This immortal monocyte cell line shows proliferation in vitro and is, therefore, suitable for immunological studies, unlike isolated human blood cells. Stimulation of THP-1 cells with phorbol-12-myristate-13-acetate (PMA) leads to their differentiation into active macrophages and thus recreates the body’s own immune response upon contact with pathogens [20]. In particular, PMA activates protein kinase C, which regulates signal transduction into the target cell through phosphorylation and triggers cell differentiation via p21 target gene activation [21]. As a result, the cells become adherent and stop their proliferation [22]. The functional and morphological properties such as shape, structure, cell surface characteristics or differentiation markers are similar to those of primary macrophages. Thus, they represent a suitable model to study human LPS-induced inflammation and pro-inflammatory cytokine release, as well as in vivo phytopharmaceutical effects, similar to the previously mentioned α-humulene effects [23, 24].

## Materials and methods

All chemical components were purchased from Sigma-Aldrich (St. Louis, Missouri, USA) unless stated otherwise.

### THP-1 cell culture and differentiation

THP-1 cells were purchased from the German Collection of Microorganisms and Cell Cultures (ACC 16, Leibniz Institute DSMZ; Brunswick, Germany) and cultivated in a humidified incubator (ICO 150, Memmert; Schwabach, Germany) at 37 °C and 5% CO_2_ in T-flasks. The cells were maintained at a cell concentration of 5*10^5^ cells/mL and sub-cultivated every 2–3 days by addition, or medium exchange after centrifugation (90 x *g*, 10 min), of fresh and pre-warmed RPMI-1640 medium, which was composed of the following components: RPMI-1640 supplemented with 2 mM L-glutamine (art. no. R8758), 10% (v/v) heat-inactivated fetal bovine serum (art. no. F9665), and 10 U/mL penicillin-streptomycin solution (art. no. P0781). The THP-1 cell count was determined using a Neubauer improved counting chamber (0640010, depth 0.1 mm, 0.0025 mm^2^ Bright-Line; Paul Marienfeld, Lauda-Königshofen, Germany). Furthermore, cell viability was monitored using a trypan blue staining assay (art. no. 93595). Cell counting and trypan blue staining was only performed during the growth phase of the THP-1 cells to allow appropriate sub-cultivation of the suspension cells prior to differentiation and the start of the study.

When the total cell number reached 12*10^6^ cells, the THP-1 cells were centrifuged (90 x *g*, 10 min) and the pellet was resuspended in 24 mL of a fresh RPMI-1640 medium containing 100 nM PMA (art. no. P8139) to induce monocyte-macrophage differentiation. For this purpose, the PMA stock used was previously dissolved in dimethyl sulfoxide (art. no. D2650) and diluted in 1x phosphate buffered saline buffer (PBS, art. no. D1408). Subsequently, 1 mL of this cell suspension (5*10^5^ cells/mL) was added to each well of a 24-well tissue plate and incubated for a differentiation time of 48 h.

### Stimulation of activated macrophages

After 48 h of PMA differentiation, non-attached cells and the medium containing PMA were removed to enhance further differentiation, the adherent macrophage-like cell layer was washed twice with 1x PBS, and 500 µL of fresh complete medium was added. LPS from *Escherichia coli* (art. no. L4391), dissolved in 1x PBS, were applied at a final concentration of 5 ng/mL into the associated wells to initiate the inflammatory cell response. After 2 h, the α-humulene stimuli (art. no. PHL83351), as well as hydrocortisone (HC, art. no. H6909) as positive controls, and alcohol (96% EtOH) as negative controls, were added according to Table 1. The α-humulene was previously diluted in 96% EtOH, and all stimuli were added to a total volume of 10 µL in the specific wells. Following 24 h of stimuli exposure during incubation at 37 °C and 5% CO_2_, the supernatants from each well were collected, vortexed, aliquoted, and frozen at −80 °C until cytokine analysis.

**Table 1:** Overview of tested stimuli with their concentrations, PMA differentiated THP-1 cells, performed in 24-well tissue plates

Basis	Stimuli					
5 ng/mL LPS	0.5 µM HC	0.25 µM HC	0.5 µM α-humulene	100 µM α-humulene	1000 µM α-humulene	2% (v/v) 96% EtOH
no LPS	0.5 µM HC	0.5 µM α-humulene	100 µM α-humulene	1000 µM α-humulene	2% (v/v) 96% EtOH	

### Analysis of pro-inflammatory cytokines TNF-α, IL-6, and IL-1β by enzyme-linked immunosorbent assay (ELISA)

Quantitative analysis of the cytokines TNF-α, IL-6, and IL-1β was performed using the ELISA Ready-SET-Go!™ kits (art. no. TNF-α - 15521127, IL-6 – 15531037, and IL-1β - 15541087) obtained from Thermo Fisher Scientific (Waltham, Massachusetts, USA). All assays were performed according to the manufacturer’s protocol using 1 M H_2_SO_4_ (art. no. 1.60313) as a stop solution. Finally, the plates were analysed with the microplate reader Infinite 200Pro (Tecan, Männedorf, Switzerland) according to the following parameters: mode absorption, measuring wavelength 450 nm, reference wavelength 570 nm, bandwidth 9 ms, number of flashes 20, rest time 0 ms, plate geometry: Costar 96 flat bottom transparent, and temperature 22–25 °C using software version Tecan i-control 1.10.4.0.

### Light microscopic imaging of THP-1 cells in suspension and after PMA differentiation into macrophages

THP-1 cell counting and viability tests were performed using 100x magnification light microscope (type 11090137002; Leica Microsystems CMS GmbH, Wetzlar, Germany). Verification of the PMA differentiation achieved in the 24-well plate, as well as the generation of images of macrophage-like cells, were also performed by light microscopy (DMi1 and type DMI6000 B; Leica Microsystems CMS GmbH, Wetzlar, Germany) using Leica Application Suite 4.12.0.

### Statistical analysis

The absorbance values subtracted from their reference wavelengths were used as surrogate parameters to determine the final cytokine concentrations. All experiments were repeated three times (*n* = 3), and the data are presented as mean ± standard deviation. GraphPad Prism 9.0.0 software was used to present results and test for significance. A one-way analysis of variance (ANOVA) followed by Dunnett’s test for multiple comparisons (95% confidence interval) was performed. Differences between stimuli and/or controls were considered significant at a level of *p* ≤ 0.05 for all tests.

## Results

### Light microscopic imaging of PMA-differentiated THP-1 cells

**Fig. 1:**
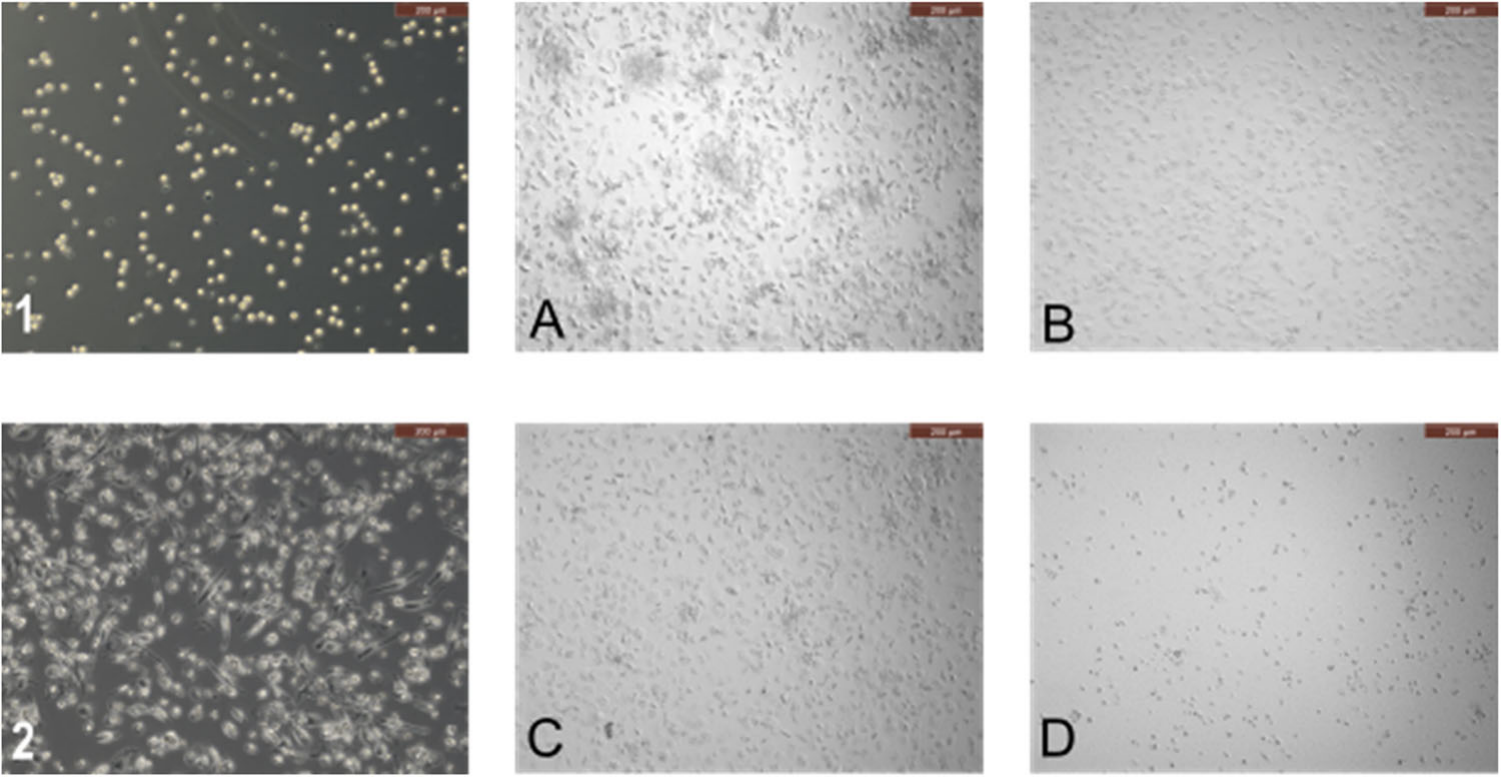
Light microscopic images of THP-1 cells and adherent macrophage-like cells after PMA differentiation of THP-1 cells and addition of stimuli, differentiation with 100 nM PMA & 48 h exposure time in 24-well plates. (1) THP-1 monocytes in cell suspension. (2) Macrophage-like cells after differentiation of THP-1 cells with PMA. **A** Macrophage-like cells after 24 h exposure to 5 ng/mL LPS. **B** Macrophage-like cells after 24 h exposure to 5 ng/mL LPS + 0.5 µM α-humulene. **C** Macrophage-like cells after 24 h exposure to 5 ng/mL LPS + 100 µM α-humulene. **D** Macrophage-like cells after 24 h exposure to 5 ng/mL LPS + 1000 µM α-humulene. The scale bar equals 200 µm

The PMA-induced differentiation of THP-1 cells into macrophage-like cells was examined and confirmed by light microscopy (Fig. 1). Furthermore, the effect of 0.5, 100, and 1000 µM α-humulene concentrations in combination with the application of 5 ng/mL LPS on the morphological characteristics of PMA-differentiated THP-1 cells was investigated (Fig. 1A–D).

Exposure to 100 nM PMA for 48 h changed the morphology of THP-1 suspension cells from previously round and non-adherent to plastic-adherent and macrophage-like (Fig. 1 1–2). This phenotype was not affected by the addition of 5 ng/mL LPS or the further 0.5 and 100 µM α-humulene addition for 24 h, respectively (Fig. 1A–C). However, after the addition of 1000 µM α-humulene, it was observed that the adherent cells were disrupted after an exposure time of 24 h, the medium became turbid and small droplets formed. After addition of 1000 µM α-humulene following LPS induction, no more adherent macrophage-like cells were found at the bottom of the 24-well plate, only cell fragments were visible as dark dots (Fig. 1D). In summary, THP-1 derived macrophage-like adherent cells were generated by differentiation using PMA, which disintegrated after the addition of 1000 µM α-humulene.

### TNF-α cytokine release in PMA differentiated THP-1 cells after the addition of stimuli

**Fig. 2:**
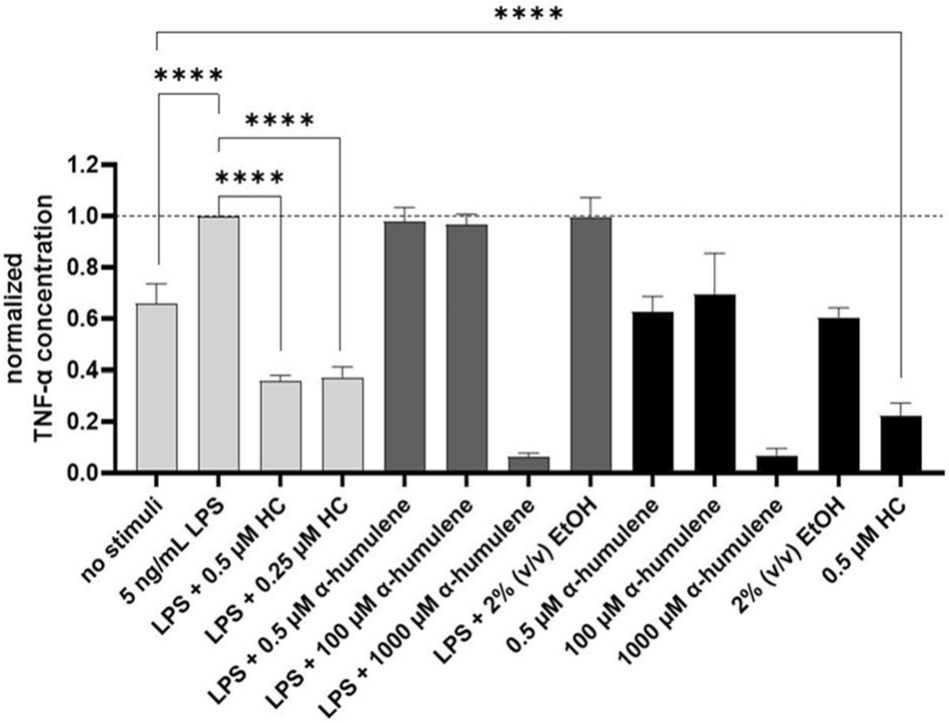
TNF-α concentration normalized on LPS addition after stimulation of PMA-differentiated THP-1 cells, 24 h exposure, statistical analysis using one-way ANOVA (*n* = 3), ****p ≤ 0.0001

To confirm a possible anti-inflammatory effect after the addition of α-humulene stimuli to PMA-differentiated THP-1 cells, the released cytokine concentrations of TNF-α, IL-6, and IL-1β were analysed from the cell supernatant by ELISA. The resulting TNF-α concentrations were compared and normalized on the mean value after the addition of 5 ng/mL LPS to the differentiated cells. This allows a direct comparison of anti-inflammatory effects by specific stimuli addition on LPS-induced cytokine release. The following graph (Fig. 2) demonstrates the calculated TNF-α mean values after stimuli exposure and their statistical significance.

TNF-α release was significantly increased (****p ≤ 0.0001) by the addition of 5 ng/mL LPS to the previously PMA-differentiated THP-1 cells (Fig. 2). The addition of 0.5 or 0.25 µM HC as a positive control significantly decreased (****p ≤ 0.0001) the TNF-α cytokine released concentration. Addition of 0.5–1000 µM α-humulene did not decrease the TNF-α release in comparison. Moreover, after the LPS induction and treatment with 1000 µM α-humulene, only 6% of the normalized TNF-a concentration (reference sample: LPS induction without additional stimuli – 2689 pg/mL TNF-α) was detected in the sample supernatant. In summary, TNF-α release was reduced by the addition of the positive control HC (0.5 and 0.25 µM), but the addition of 0.5 or 100 µM α-humulene did not result in a significant TNF-α reduction.

### IL-6 cytokine release in PMA differentiated THP-1 cells after the addition of stimuli

**Fig. 3:**
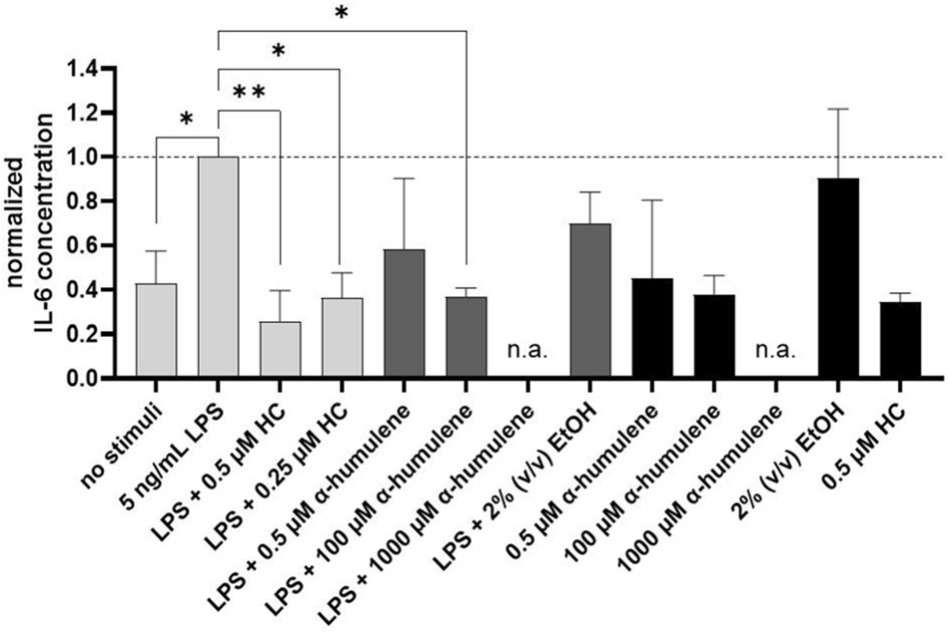
IL-6 concentration normalized on LPS addition after stimulation of PMA-differentiated THP-1 cells, 24 h exposure, statistical analysis using one-way ANOVA (*n* = 3), **p ≤ 0.01

Identical to the previous TNF-α investigation, the IL-6 cytokine concentration was also determined in the cell supernatant by ELISA. Here, IL-6 concentrations, after the addition of different stimuli, were compared with respect to the normalized value (addition of 5 ng/mL LPS). The following graph (Fig. 3) shows the calculated IL-6 levels after exposure to the stimuli and their statistical significance.

IL-6 release was significantly increased (*p ≤ 0.05) by the addition of 5 ng/mL LPS to the previously PMA-differentiated THP-1 cells (Fig. 3). The addition of 0.5 (**p ≤ 0.01) or 0.25 µM HC (*p ≤ 0.05) as a positive control significantly decreased the IL-6 cytokine concentration released. Addition of 0.5 µM α-humulene did not significantly decrease IL-6 release in comparison, whereas the IL-6 cytokine concentrations after treatment with 1000 µM α-humulene could not be detected and were below the detection limit (n.a.). In addition, after the LPS induction and treatment with 100 µM α-humulene, IL-6 concentration could be significantly reduced (* *p* ≤ 0.05) to 37 ± 4% of the normalized reference value (LPS induction without additional stimuli – 20 pg/mL IL-6). Overall, IL-6 release was reduced in a dose-dependent manner by addition of the positive control HC (0.5 and 0.25 µM), and the addition of 100 µM α-humulene led to a significant IL-6 reduction.

### IL-1β cytokine release in PMA-differentiated THP-1 cells after the addition of stimuli

**Fig. 4:**
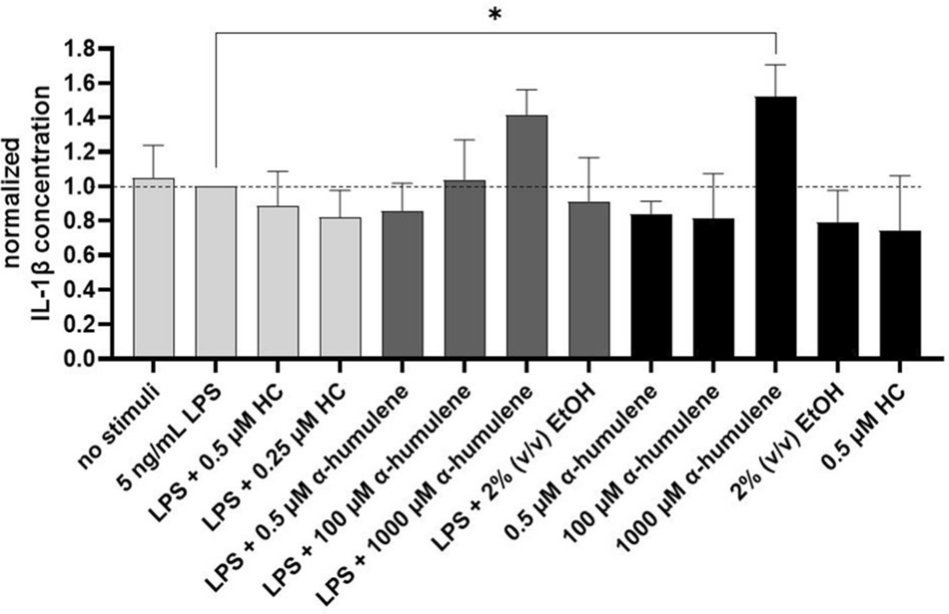
IL-1β concentration normalized on LPS addition after stimulation of PMA-differentiated THP-1 cells, 24 h exposure, statistical analysis using one-way ANOVA (*n* = 3), *p ≤ 0.05

As a third pro-inflammatory cytokine, levels of IL-1β release were determined identically as described before by ELISA. The IL-1β concentrations after the addition of different stimuli were compared with respect to the normalized value (5 ng/mL LPS addition). The graph below (Fig. 4) shows the IL-1β values after the exposure to stimuli and the statistical significance.

No significant increase in IL-1β cytokine release was detected by the addition of 5 ng/mL LPS to the previously PMA-differentiated THP-1 cells (Fig. 4). The addition of 0.5 or 0.25 µM HC as a positive control hardly reduced the LPS-induced IL-1β release compared to the previous cytokines. All stimuli additions resulted in IL-1β concentrations at the normalized reference level considering their standard deviations. However, it can be seen that the addition of 1000 µM α-humulene resulted in a significantly higher IL-1β release (*p ≤ 0.05) compared to the normalized reference value (LPS induction without additional stimuli – 174 pg/mL IL-1β) or the control without stimuli. In summary, IL-1β release was not significantly altered by the addition of the stimuli tested compared with the control value (addition of 5 ng/mL LPS). However, the addition of 1000 µM α-humulene significantly increased the release of IL-1β, while the addition of 1000 µM α-humulene resulted in lower to undetectable TNF-α and IL-6 cytokine concentrations.

## Discussion

The results of this study demonstrate, until date, for the first time that a treatment with the sesquiterpene α-humulene can decrease LPS-induced release of the pro-inflammatory cytokine IL-6 in a dose-dependent manner. Of particular interest is the fact that the study was carried out using the human monocyte cell line THP-1, which has been differentiated into activated macrophages, allowing direct translation of the findings to human inflammatory responses, in contrast to many animal cell models. The addition of 5 ng/mL LPS to PMA-differentiated THP-1 monocytes resulted in significantly increased TNF-α and IL-6 cytokine concentrations. Since LPS belongs to the class of pathogen-associated molecular patterns, it is immunologically active and binds to surface structures such as pattern recognition receptors. These LPS-binding proteins can now transport the binding complex to the membrane-bound CD14 surface protein, found on monocytes and macrophages. After further interactions with several membrane receptors, it is assumed that toll-like receptors 4 bind this complex and transmit the signal intracellularly via dimerization with the help of the mediator protein MD-2. There, it leads to the activation of the NF-κB signaling pathway and an increased release of various cytokines by further, partly unknown, mechanisms. This cytokine conglomerate also includes the pro-inflammatory cytokines TNF-α, IL-6, and IL-1β tested in this study [25, 26].

This increased LPS-induced TNF-α and IL-6 cytokine release, which was significant in the results, could be inhibited in a dose-dependent manner by the addition of 0.5 and 0.25 µM HC, respectively. HC was used in this study as a pharmacologically established and effective positive control, which initiates an anti-inflammatory effect by counteracting the LPS-induced activation of the NF-κB pathway to a certain extent [27]. In doing so, HC induces transcription of the NF-κB cytoplasmic inhibitor protein gene IκBα, which decreases the quantity of NF-κB by binding and deactivation in the cytoplasm and preventing NF-κB from entering the nucleus [28]. An anti-inflammatory effect was also successfully observed after the addition of 100 µM α-humulene, which showed a significant decrease of LPS-induced IL-6 release by 60% that can be compared to the anti-inflammatory effect of a 0.25 µM HC dosage in this study. The addition of 0.5 µM α-humulene after LPS induction also decreased IL-6 release, to a lesser extent, suggesting a dose-dependent pattern of effect. How α-humulene affects the inflammatory cascade and has an anti-inflammatory effect has not been studied in detail. Studies have shown a decrease in NF-κB activation and reduced neutrophil migration in the tissue of LPS-inflamed rat paws after α-humulene addition [29].

Giving α-humulene to the inflamed lung tissue of female BALB/c mice also decreased NF-κB activation and activator protein 1 [30]. Furthermore, a decrease of IL-6 mRNA expression levels and histamine levels, via control of intracellular calcium and cyclic adenosine monophosphate (cAMP) levels, in PMA-stimulated human mast cells HMC-1 cells was observed by adding α-humulene [31]. In paw oedema of rats previously inflamed by carrageenan injection, anti-inflammatory effects and a down-regulation of prostaglandin E2 by α-humulene addition were also demonstrated [32].

These effects of α-humulene described in the literature may all interact to produce the IL-6 inhibition shown in this study. NF-κB and activator protein 1 signaling play major roles in the initiation of inflammation in human cells through their transcriptional activities, and weakening the binding affinity of their transcription factors to cytokine receptors leads to additional anti-inflammatory effects [33]. Thus, this reduced activity by α-humulene successively decreases IL-6 release in the PMA-stimulated THP-1 cells used, as it has also been shown in the literature after administration of epimagnolin A by attenuated IL-6 promoter activity [34]. Since histamine additionally upregulates IL-6 release in M1 macrophages, histamine levels lowered by α-humulene, as previously described, thus directly influences IL-6 release in the demonstrated dose-dependent manner [35]. The modulator of the immune response, prostaglandin E2, induces IL-6 expression in humans via cAMP/PKA and PI3K-dependent pathways and its NF-κB activation coupled with NF-κB subunit p65 binding to the IL-6 promotor [36]. By lowering prostaglandin E2 levels and controlling intracellular cAMP levels, α-humulene effectively inhibits IL-6 synthesis [36, 37].

Other sesquiterpenoids have also been shown to have anti-inflammatory properties via LPS-induced IL-6 cytokine release: zerumbone - IC_50_ of 2.5 μM in THP-1 derived macrophages [38], popolohuanones G-I - 73.1% inhibitory activity at 10 µM in THP-1 cells [39], chlorojanerin - IC_50_ of 1.8 ± 0.7 μM in THP-1 cells [40], estafiatin - IC_50_ of 3 μM in THP-1 cells [41]. Some monoterpenes may also have anti-inflammatory effects. Here, it was shown that the administration of 10 µM 1,8-cineol, which is successfully used as a drug component against human respiratory diseases [42], reduced LPS-induced IL-6 secretion in isolated human monocytes by 76 ± 10% [43]. In LPS-stimulated RAW 264.7 cells, administration of 0.04% D-limonene (2470 µM) reduced IL-6 secretion by 80% in a dose-dependent manner [44]. Moreover, toxic effects were also observed after the addition of an excessive α-humulene concentration (1000 µM). Toxicity studies showed a cytotoxic concentration that reduces cell viability by 50% (CC_50_ value) of two *Salvia officinalis* sage extracts, which are rich in α-humulene, on differentiated THP-1 macrophages between 66.70 ± 5.41 and 80.24 ± 7.58 µg/mL, respectively, after 24 h exposure [45]. Furthermore, a CC_50_ value of 109.7 ± 2.3 μg/mL and 29.0 ± 0.3 μg/mL, respectively, was demonstrated when lineage Vero and HeLa cells were stimulated with α-humulene for 72 h [46]. The CC_50_ values found in literature confirm the assumption that cytotoxic effects and cell lysis, as observed by light microscopy, occurred in this study at the α-humulene concentration of 1000 µM (equals 204 µg/mL), which is clearly above the CC_50_ values mentioned above. Due to this early-stage macrophage cell lysis, hardly any TNF-α or IL-6 cytokines were expressed and released via their cascade, which explains the low or undetectable TNF-α or IL-6 cytokine concentrations after addition of 1000 µM α-humulene. The release of IL-1β, on the other hand, was increased by addition of 1000 µM α-humulene above the normalized LPS reference value in this study. This initially contradictory finding, can be explained by the intracellular uptake and storage of IL-1β in activated macrophages, whose function is to activate and release small IL-1β amounts during an inflammatory reaction [47]. Thus, as a consequence of cell disintegration by toxic amounts of α-humulene (as shown in this study with 1000 µM α-humulene), there may be an uncontrolled release of the intracellularly stored IL-1β, resulting in high IL-1β titres as shown in the results [48, 49]. The reduction of pro-inflammatory IL-6 levels by α-humulene shown in this study has enormous immunoregulatory benefits for the human organism. This is because IL-6 titres play a key role in molecular signaling and are significantly involved in the initiation of acute phase reactions in inflammatory processes [50].

Furthermore, elevated IL-6 levels also negatively influence the ongoing clinical process, as titres above 24 pg/mL have been shown to induce hypoxemia in Covid-19 patients [51]. In addition, chronically elevated IL-6 levels are associated with the formation and growth of several tumors such as breast cancer [52], making IL-6 a cancer marker, leading to a great need for novel IL-6-lowering cancer immunotherapy approaches [53, 54].

In conclusion, the addition of 0.5 and 100 µM α-humulene had anti-inflammatory effects on LPS-induced IL-6 release in differentiated THP-1 cells, while α-humulene did not affect the release of TNF-α and IL-1ß at these concentrations in our study. Thus, our findings could be transferred to the human organism to provide α-humulene as an alternative, natural-based, gentle and promising therapeutic approach against elevated IL-6 levels and chronic inflammation. Which consequently lead to further research in the application of α-humulene.

## Data Availability

The data presented in this study are available on request from the corresponding author.
